# A Case of Multifocal Burkitt Lymphoma in an Immunocompromised Patient

**DOI:** 10.7759/cureus.32183

**Published:** 2022-12-04

**Authors:** Ariana R Tagliaferri, Wadah Akroush, Abdalla Mohamed, Walid Baddoura, Yana Cavanagh

**Affiliations:** 1 Internal Medicine, St. Joseph's Regional Medical Center, Paterson, USA; 2 Internal Medicine, St. Joseph's University Medical Center, Paterson, USA; 3 Medicine, St. Joseph's University Medical Center, Paterson, USA; 4 Gastroenterology, St. Joseph's Regional Medical Center, Paterson, USA

**Keywords:** primary gastro intestinal lymphoma, gastric tumor, b cell neoplasm, non hodgkin's lymphoma, sporadic burkitt lymphoma

## Abstract

Non-Hodgkin lymphoma is made from the B-cell lineage and includes extra-nodal marginal lymphomas, follicular lymphomas, mantle cell lymphoma, diffuse large B-cell lymphoma, and Burkitt lymphoma. Burkitt lymphoma is associated with Epstein Barr Virus and Human Immunodeficiency Virus. Although it is common for other B-cell lymphomas to develop in the stomach, it is less common for Burkitt lymphoma tumors to manifest there. Additionally, primary and/or secondary involvement of the duodenum, pancreas, and intestines is very rare in Burkitt lymphoma. Herein, we present a male diagnosed with extensive Burkitt lymphoma of the bone, lymph nodes, pancreas, small intestine, duodenum, and stomach.

## Introduction

Non-Hodgkin lymphomas (NHL) are a type of lymphatic tissue malignancy made from the B-cell lineage [1,2]. B-cell malignancies are comprised of either extra-nodal marginal lymphoma, follicular lymphomas, mantle cell lymphoma, diffuse large B-cell lymphoma, or Burkitt lymphoma (BL) [2]. Of these, diffuse large B-cell lymphoma is the most common histological subtype, and only 1%-2% of NHL are BL [2,3]. BL is more common in Caucasians and more prevalent in males [4]. It is the eighth most common cancer in men and the 11^th^ most common cancer in women, accounting for approximately 4% of all cancers [3].

BL was first described in the mid-1900s in tropical Africa as a sarcoma in children [5]. By 1961, a British pathologist discovered a virulent strain within the tumor tissue for the first time in history, which he called the Epstein Barr Virus (EBV) [5]. Since then, it has been widely recognized that BL is associated with EBV, although the pathogenesis is not well understood [4,5]. It is thought that EBV interferes with normal gene expression and translation of microRNA [5]. Today, BL is also associated with Human Immunodeficiency Virus (HIV), accounting for up to 40% of all lymphomas in HIV patients [4,5]. There are three types of BL: endemic, which is prevalent in Africa and Papua New Guinea, primarily in children, sporadic localized to North America and Europe and immunodeficiency-associated, which occurs globally [4,5]. EBV is linked to all three types of BL [5].

The translocation t(8:14) from the c-MYC gene is observed in approximately 80% of cases and is considered the hallmark of the disease, although the presence of MYC rearrangement is not specific for BL [4]. Histologically, there are mature lymphocytes with round nuclei with basophilic cytoplasm, prominent vacuoles, and highly proliferative [4]. They also have a “starry sky” appearance on hematoxylin-eosin staining due to the increased number of mitotic figures and cytoplasmic borders [1,4]. Ki67 positivity reflects the rapid proliferation rates, approaching 100% [1,4]. On immunohistochemistry, the B-cell markers will be positive for CD19, CD20, CD79a, CD10, PAX5, BCL-6, and EBV-associated forms will also express CD21 [1,4].

Although diffuse large B-cell lymphoma commonly affects the gastrointestinal tract and colon, it is less common for Burkitt lymphoma to manifest there [2]. Endemic BL manifests in the bones of the jaw, face, kidneys, ovaries, and breasts, and occasionally the gastrointestinal tract, whereas Sporadic BL most commonly affects the ileocecal region, ovaries, kidneys, omentum, and central nervous system [5]. Constitutional symptoms are frequently reported [4]. Gastric lymphoma is most commonly found in the gastric antrum, which can manifest in the form of ulcers, heterogeneous masses, or a combination of both within multiple locations of the antrum [6]. *Helicobacter pylori *is another important cause of gastric lymphoma that needs to be excluded at the time of diagnosis [6]. Ann Arbor or Murphy staging is used most often using bone marrow samples or samples from the primary tumor if one is found [4]. Although B-cell lymphomas are aggressive due to rapid proliferation, they are responsive to chemotherapy agents such as CVP (cyclophosphamide, vincristine, prednisone), MACOP-B (methotrexate, adriamycin, cyclophosphamide, vincristine, prednisone, bleomycin), and CHOP (cyclophosphamide, doxorubicin, vincristine, prednisone, +/- rituximab) [1,5]. Poor prognostic indicators include poor response to chemotherapy, presence of cerebrovascular disease at the time of diagnosis, elevated lactose dehydrogenase levels, and deletion of 13q or gain of 7q on cytogenetic workup [4].

## Case presentation

A 52-year-old African-American male with a past medical history of the human immunodeficiency virus (HIV) and Acquired Immune Deficiency Syndrome (AIDS), untreated Hepatitis C, and polysubstance use with intravenous drug abuse (IVDA) presented with left-sided back pain radiating to the stomach for two weeks before admission. The pain was sharp, exacerbated with movement, and improved with rest. He denied any inciting events or trauma to the area. Their blood pressure was 98/58 mmHg, heart rate was 81 beats per minute, respiratory rate was 18 breaths per minute, and he was saturating 100% on room air. On physical examination, the patient appeared cachectic with temporal wasting, track marks were present on the right arm, and he was lying on his right side in acute distress due to pain. The patient demonstrated severe abdominal pain to deep and superficial palpation throughout all quadrants and had focal tenderness upon palpation of his lumbar spine and left paraspinal muscles. Laboratory findings were most significant for relative leukocytosis, normocytic anemia, acute kidney injury (AKI), elevated alkaline phosphatase, elevated lactate dehydrogenase (LDH), and erythrocyte sedimentation rate (ESR), and urine toxicity positive for cocaine and opiates (Table 1).

**Table 1:** Admission Laboratory Values With Reference Ranges. A comprehensive metabolic panel and complete blood count demonstrate abnormalities in kidney function, electrolytes, albumin, alkaline phosphatase, and hemoglobin.

Complete Metabolic Panel	Values (Reference Ranges)	Complete Blood Count	Values (Reference Ranges)
Sodium	133 mEq/L (135-145mEq/L)	White Blood Cells	7.8x10^3/mm3 (4.5-11.0x10^3/mm3)
Potassium	5.0 mEq/L (3.5-5.0mEq/L)	Red Blood Cells	4.20 x10^6/mm3 (4.33-5.83x10^6/mm3)
Chloride	101 mEq/L (98-107 mEq/L)	Hemoglobin	11.1g/dL (13.5-17.5g/dL)
Carbon dioxide	24 mEq/L (21-31 mEq/L)	Hematocrit	34.5% (41.0-53.0%)
Glucose	101 mg/dL (70-110 mg/dL)	MCV	82.1 fL (80-100 fL)
Blood urea nitrogen	61mg/dL (7-23mg/dl)	MCH	26.4 pg (26-32 pg)
Creatinine	4.14mg/dL (0.60-1.30mg/dl)	MCHC	32.2 g/dL (31-37 g/dL)
Calcium	8.5mg/dL (8.60-10.30mg/dl)	RDW	14.0% (0.5-16.5%)
Albumin	2.7 g/dL (3.5-5.7 g/dL)	Platelet	315 K/mm3 (140-440 K/mm3)
Alkaline phosphatase	261 unit/L (34-104 unit/L)		
ALT	35 unit/L (7-52 unit/L)		
AST	26 unit/L (13-39 unit/L)		
Lipase	144 unit/L (11-82 unit/L)		

An admission chest radiograph (CXR) was unremarkable. Computed Tomography (CT) of the abdomen and pelvis without contrast showed a fracture in the left ninth rib with soft tissue density extending to the left lateral epidural space, as well as a prominent gastric wall thickening with an asymmetric appearing stomach. A Magnetic Resonance Imaging (MRI) of the thoracic (T) spine without contrast showed abnormal marrow signals in multiple thoracic vertebrae highly suspicious for multifocal osteomyelitis (OM) with epidural abscess spanning from vertebrae T7-T9.

**Figure 1:**
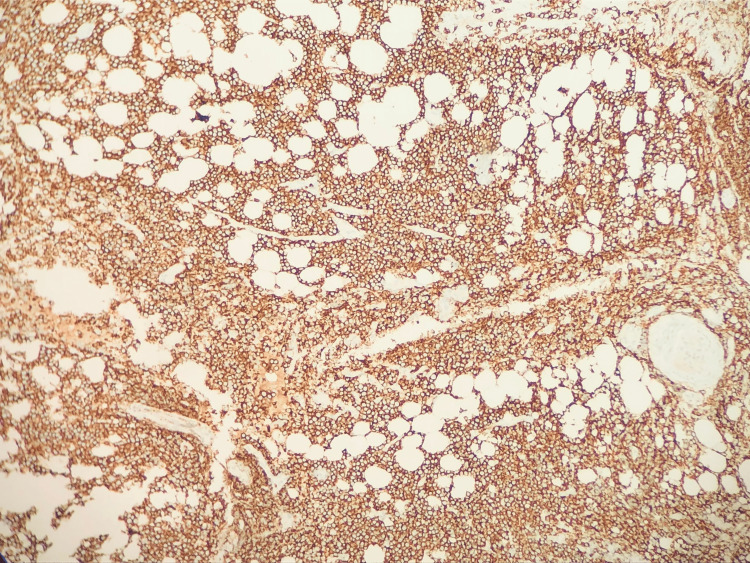
Epidural Biopsy. Epidural biopsy immunohistochemical stain positive for CD20 B-cells.

The patient was taken the next day to the operating room for incision and debridement with laminectomy. Pathology samples obtained from the epidural abscess resulted in High-Grade Diffuse B-cell Lymphoma (Figure 1).

**Figure 2:**
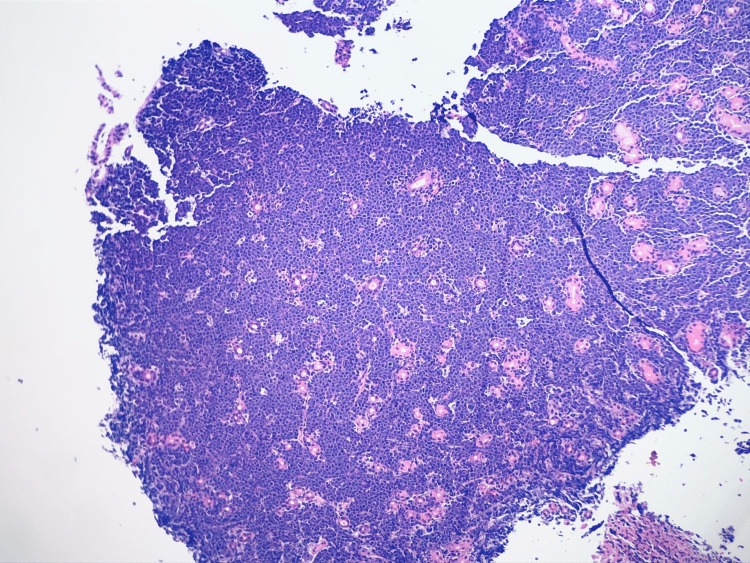
Duodenal Biopsy. Duodenal biopsy demonstrating necrosis and lymphocytic and macrophages infiltration on H&E stain.

**Figure 3:**
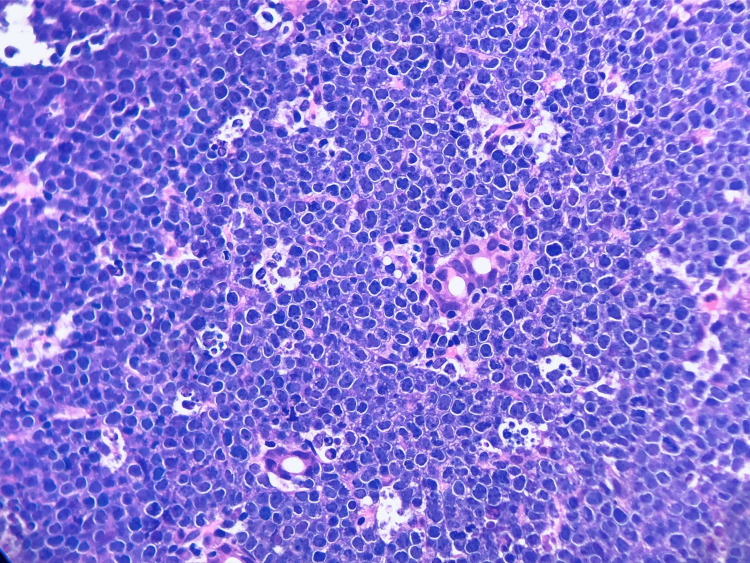
Starry Sky Pattern. Duodenal biopsy demonstrating starry sky pattern on H&E stain.

**Figure 4:**
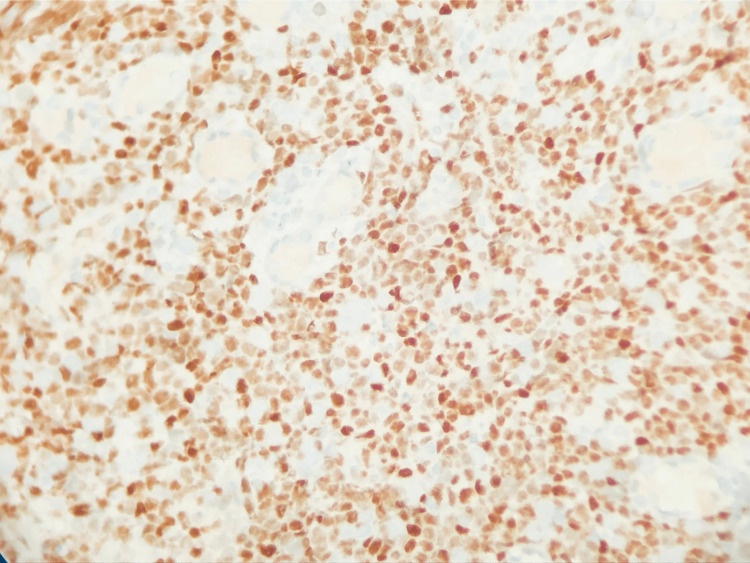
C-myc Positive Duodenum. Stain for C-myc positivity from the duodenum.

An esophagogastroduodenoscopy (EGD), endoscopic ultrasound (EUS), and endoscopic retrograde cholangiopancreatography (ERCP) were performed for the abnormal findings on earlier CT imaging, as well as rising bilirubin and liver function enzymes. Endoscopic and endosonographic findings included esophageal candidiasis, multiple masses and nodules throughout the stomach and duodenum with abutment into the liver, dilation of the common bile duct (13 mm) with stricture at the lower third of the main bile duct due to large pancreatic mass (25 mm), and pancreatic duct dilation (4 mm) with another mass visualized in the pancreatic body (15 mm). A biliary sphincterotomy was performed. Additionally, there were numerous lymph nodes in the celiac and peri-gastric regions. All biopsies of the stomach, duodenum, lymph nodes, and pancreatic body were positive for Burkitt's lymphoma (BL) with Epstein-Barr virus-positive EBV+ (Figures 2-4).

A biopsy of the pancreatic head mass was negative for malignancy. To determine staging, a CT of the chest, bone scan, cerebrospinal fluids (CSF) flow cytometry, and MRI of the brain and spine were performed. Bone scan showed increased uptake in the distal shaft of the left humerus with a diffuse area of increased uptake in the midshaft of the left femur. The CT of the chest demonstrated a 4mm lung nodule, and CSF flow cytometry and MRI brain were negative for CNS involvement. The MRI of the spine, however, was positive for multifocal signal abnormalities in the thoracolumbar spine and paraspinal region suggestive of lymphoma. Oncology diagnosed him with Stage IV Burkitt Lymphoma, and further workup was obtained (Table 2).

**Table 2:** Malignancy Workup. Miscellaneous laboratory studies were sent during the workup for malignancy.

ESR	114 mm/hr (0-10 mm/hr)	CRP	16.6 mg/L (<= 9.9 mg/L)
LDH	483 unit/L (140-271 unit/L)	CA 19-9	24 Unit/mL (0-35 Unit/mL)
HCV ab	10.3 s/co (0-0.9 s/co)	HIV PCR RNA	190 copies/ml
HepC Qn.	1640000 IntlUnit/mL	CD4 count	114 /mcL (350-1519 /mcL)
Hep B Surf Ab Ql.	Reactive	Hep B Core Total Ab	Negative

On Hospital day eight, the patient became severely constipated, and an abdominal X-ray revealed multiple dilated small bowel loops suggestive of obstruction. Through a repeat CT scan, it was presumed that the obstruction was secondary to a lymphoma of the intestines, and thus chemotherapy was initiated with Cyclophosphamide, Doxorubicin, Vincristine, and Methotrexate/Rituximab, Ifosfamide, Etoposide, and Cytarabine regimen (R-CODOX/ R-IVAC). Granulocyte colony-stimulating factor (G-CSF) and Intrathecal Methotrexate were also administered. The obstruction was resolved, and the patient was sent home for further management of his Burkitt Lymphoma under the oncology team.

## Discussion

Primary gastric lymphoma is more prevalent in men older than 50 years of age and is usually due to B-cell lymphomas; however, other primary gastric lymphomas can also include mantle cell and marginal zone B-cell lymphoma (MALT) [7]. Primary non-Hodgkin’s lymphoma of the GI tract accounts for less than 0.9% of all gastrointestinal tumors [2]. Patients with primary non-Hodgkin’s lymphoma of the GI tract typically have a history of inflammatory bowel disease or radiation [2]. Generally, up to 30%-40% of extra-nodal manifestations of all non-Hodgkin lymphomas occur in the stomach; however, this is usually due to secondary involvement, especially in non-endemic BL [5,7]. Primary BL of the GI tract is very rare, and GI symptoms predominate in these patients [5]. Moreover, gastric, duodenal, and pancreatic involvement is much less common than intestinal involvement [1,3,5]. Pancreatic and duodenal involvement accounts for less than 1% of NHL tumor growth, even in secondary disease [1,3]. The incidence of pancreatic involvement in BL specifically is not known because it is so rare, and the only literature is from pediatric cases [1]. It is unknown if our patient had primary or secondary involvement of his gastrointestinal tract because he was found to have pancreatic, duodenal, gastric, lymphatic, and bone tissue positive for Burkitt Lymphoma. Additionally, he had both symptoms of gastric and colonic obstruction as well as pain in the abdomen and back, localized to the side of the malignancy. B-cell lymphoma of the colon is present in less than 0.5%; however, BL is rarer [2]. If found, patients typically present with intestinal obstruction warranting surgery. "Although our patient presented similarly with intestinal obstruction secondary to colonic lymphoma, he was treated with chemotherapy successfully to reduce tumor burden. Lastly, when BL is linked to HIV, patients typically have a CD4 count greater than 200 without concomitant opportunistic infections [4]. Our patient’s CD4 count was 114, and he was actively infected with esophageal candidiasis. This makes our case particularly unique. 

## Conclusions

In summary, we present an HIV patient with low CD4 counts who presented with localized pain and intestinal obstruction and was diagnosed with Burkitt lymphoma of the bone, lymph nodes, duodenum, stomach, pancreas, and small intestine. The primary site of Burkitt lymphoma is ultimately unknown due to its complex presentation; however, primary and/or secondary involvement of the duodenum, pancreas, stomach and intestine is very rare in non-endemic Burkitt lymphoma.
